# Experimental mouse model of tuberculous uveitis

**DOI:** 10.1186/1471-2334-14-S3-O20

**Published:** 2014-05-27

**Authors:** Sudhanshu Abhishek, Amod Gupta, Indu Verma

**Affiliations:** 1 Department of Biochemistry, Post Graduate Institute of Medical Education and Research, Chandigarh, India; 2 Department of Ophthalmology, Post Graduate Institute of Medical Education and Research, Chandigarh, India

## Background

Tuberculous uveitis is one of the emerging forms of extrapulmonary tuberculosis. To understand the many unknown facets of this disease pertaining to diagnosis, treatment and pathogenesis, a mouse model of tuberculous uveitis was established.

## Methods

Mice (Balb/c) were infected with *Mycobacterium tuberculosis* (H37Rv) through two different routes, i.v. and i.n. at different doses. Delayed type hypersensitivity (DTH) assay at 24 and 48hours before sacrifice, CFU enumeration in eyes, RT-PCR, histopathological studies and cytokine analysis were performed in these animals.

## Results

The i.v. and i.n. challenged mice showed significant DTH response. The CFU enumeration in eye did not show any bacilli initially by 30th day however tubercle bacilli could be recovered from the eyes of i.v. infected mice by 45^th^ day at the time of death. Histopathology of these eyes showed inflammation with cluster of lymphocytes. The RT- PCR for IS6110 gene from the eyes of i.n. infected animals was positive at different doses whereas i.v. challenged mice showed variability. Furthermore, the cytokine analysis in infected tissue supernatants of both eyes and lung showed high Th1 response (IFN-γ) in comparison to non-infected group.

## Conclusion

Overall, this study evaluated the ability of *M. tb* to establish infection in the eyes of mouse model following infection with different routes, doses and at different time points. RT-PCR positivity for IS6110 gene along with high IFN-γ response in the eyes of infected animals as compared to non-infected mice without any bacteriological evidence indicate the establishment of a paucibacillary TB uveitis mouse model, a model mimicking the human TB uveitis condition.

